# Deep Sequencing Reveals Transcriptome Re-Programming of *Taxus × media* Cells to the Elicitation with Methyl Jasmonate

**DOI:** 10.1371/journal.pone.0062865

**Published:** 2013-04-30

**Authors:** Guiling Sun, Yanfang Yang, Fuliang Xie, Jian-Fan Wen, Jianqiang Wu, Iain W. Wilson, Qi Tang, Hongwei Liu, Deyou Qiu

**Affiliations:** 1 Key Laboratory of Economic Plants and Biotechnology, Kunming Institute of Botany, Chinese Academy of Sciences, Kunming, China; 2 State Key Laboratory of Tree Genetics and Breeding, The Research Institute of Forestry, Chinese Academy of Forestry, Beijing, China; 3 Department of Biology, East Carolina University, Greenville, North Carolina, United States of America; 4 State Key Laboratory of Genetic Resources and Evolution, Kunming Institute of Zoology, Chinese Academy of Sciences, Kunming, Yunnan, China; 5 CSIRO Plant Industry, Canberra, Australian Capital Territory, Australia; 6 Guangxi Botanical Garden of Medicinal Plant, Nanning, Guangxi, China; The Centre for Research and Technology, Hellas, Greece

## Abstract

**Background:**

Plant cell culture represents an alternative source for producing high-value secondary metabolites including paclitaxel (Taxol®), which is mainly produced in *Taxus* and has been widely used in cancer chemotherapy. The phytohormone methyl jasmonate (MeJA) can significantly increase the production of paclitaxel, which is induced in plants as a secondary metabolite possibly in defense against herbivores and pathogens. In cell culture, MeJA also elicits the accumulation of paclitaxel; however, the mechanism is still largely unknown.

**Methodology/Principal Findings:**

To obtain insight into the global regulation mechanism of MeJA in the steady state of paclitaxel production (7 days after MeJA addition), especially on paclitaxel biosynthesis, we sequenced the transcriptomes of MeJA-treated and untreated *Taxus × media* cells and obtained ∼ 32.5 M high quality reads, from which 40,348 unique sequences were obtained by *de novo* assembly. Expression level analysis indicated that a large number of genes were associated with transcriptional regulation, DNA and histone modification, and MeJA signaling network. All the 29 known genes involved in the biosynthesis of terpenoid backbone and paclitaxel were found with 18 genes showing increased transcript abundance following elicitation of MeJA. The significantly up-regulated changes of 9 genes in paclitaxel biosynthesis were validated by qRT-PCR assays. According to the expression changes and the previously proposed enzyme functions, multiple candidates for the unknown steps in paclitaxel biosynthesis were identified. We also found some genes putatively involved in the transport and degradation of paclitaxel. Potential target prediction of miRNAs indicated that miRNAs may play an important role in the gene expression regulation following the elicitation of MeJA.

**Conclusions/Significance:**

Our results shed new light on the global regulation mechanism by which MeJA regulates the physiology of *Taxus* cells and is helpful to understand how MeJA elicits other plant species besides *Taxus*.

## Introduction

*Taxus*, also known as yew, is a genus of gymnosperm and contains at least 14 species [1]. *Taxus* can produce a number of chemicals with pharmaceutical properties. Among them, paclitaxel (Taxol®) was reported to be a very promising anticancer drug in 1964 [2] and now is used widely in chemotherapy treatment of lung, ovarian, and breast cancer [3]. Paclitaxel was originally extracted from the bark of *Taxus brevifolia* but yields are low; approximately 0.01% of the dry bark weight [4]. With growing demand for the paclitaxel for medical use, harvesting from bark cannot meet the demand and also raises serious ecological concerns.

Great efforts have been taken to increase the production of paclitaxel by finding alternative sources and methods of synthesis including *Taxus* needles [4], [5] fungal sources [4], [5], developing semisynthesis method from related taxanes [6], and using plant cell cultures [7]. Due to the high demand, a combined method of production is mostly used in which an abundant natural intermediate metabolite compound is isolated from natural sources (yew bark and needle) or by plant cell culture, and then using semi-synthesis methods to obtain paclitaxel.

Plant cell culture has the potential for producing large quantities of paclitaxel but currently suffers from low yields. Some compounds are known to stimulate the production of paclitaxel when added to the medium. Among them, methyl jasmonate (MeJA) has been found to significantly elicit the production of paclitaxel in the cultured cells of *T. × media*, *T. canadensis*, *T. cuspidata* etc. [8], [9], [10] with the greatest accumulation of paclitaxel, 36.0 mg/L, observed when the cultured cells were elicited with 200 µM MeJA [9]. However, the addition of MeJA in *Taxus* cell culture also retards cell growth [10] and therefore reduces indirectly the yield of paclitaxel in culture. A detailed knowledge of the biosynthesis of paclitaxel and its regulation by MeJA is required before directed bioengineering methods could be implemented to increase paclitaxel yields in cell culture.

Next generation sequencing technologies have been proved to be rapid and cost-effective means to analyze the genome and transcriptome in non-model species [11], [12], [13]. Improvement in *de novo* assembly of high-throughput sequencing data and relative accurate estimation of gene expression levels makes this approach also powerful in quantifying gene expression [14], [15], [16]. This technology has been applied to attempt to understand varying aspects of paclitaxel synthesis in *Taxus* [17], [18], [19]. Hao et al [17] assembled a *de novo* transcriptome of *T. mairie* and sequenced three different tissues (root, stem and leaves) using a tag-based digital gene expression system, and found a number of genes involved in tissue specific functions. Wu et al. [18] sequenced the transcriptome of *T. cuspidata* needles using 454 pyrosequencing. The early response of elicitation with MeJA has been studied by Li et al [19] who found 13,469 differentially expressed genes in *T. chinensis*, whereas Lenka et al [20] used PCR-based suppression subtractive hybridization (SSH) to find 300 differentially expressed genes between non-elicited and MeJA elicited *T. cuspidate* cell suspension.

To get a comprehensive understanding of the global regulation mechanism of MeJA on paclitaxel biosynthesis in steady state of paclitaxel production, we sequenced the transcriptomes of *Taxus × media* cells after 7 days without or with the elicitation of MeJA (Figure S1) when the maximum paclitaxel accumulation is observed [9], [10]. Comparing the gene expression profiles of these two cells revealed that 18 out of all 29 known genes in terpenoid backbone and paclitaxel biosynthesis had higher transcript levels by addition of MeJA. In addition to the increased transcript abundance of specific transcription factors and chromatin modification proteins, we found decreased transcript levels of genes involved in cell cycle, DNA replication, meiosis, and mismatch repair, which provides insights into the mechanism by which MeJA both inhibits cell growth while significantly inducing the production of paclitaxel.

## Results

### Illumina Sequencing and *de novo* Assembly of Sequence Reads in *Taxus × media*

Illumina high-throughput second generation sequencing was carried out to obtain the transcriptomes of *Taxus × media* cell cultures under nonelicited and elicited conditions. The sequencing produced 77,395,444 reads with each 75 base and with the total nucleotides (nt) of 5,804,658,300 nt (5.8 Gb): i) non-elicited *T. × media* transcriptome produced 37,323,590 reads (2.8 Gb); ii) *T. × media* elicited with MeJA produced 40,071,854 reads (3.0 Gb) nt. After removing the adaptor sequences, empty reads and low quality sequences (reads with more than 10% Q20 bases), we obtained 16,340,898 and 16,153,572 high quality reads from the nonelicited and elicited cultures, respectively. The sequence data have been submitted to NCBI Sequence Read Archive (SRA) database under the accession number SRX156706 and SRX156707.

**Figure 1:**
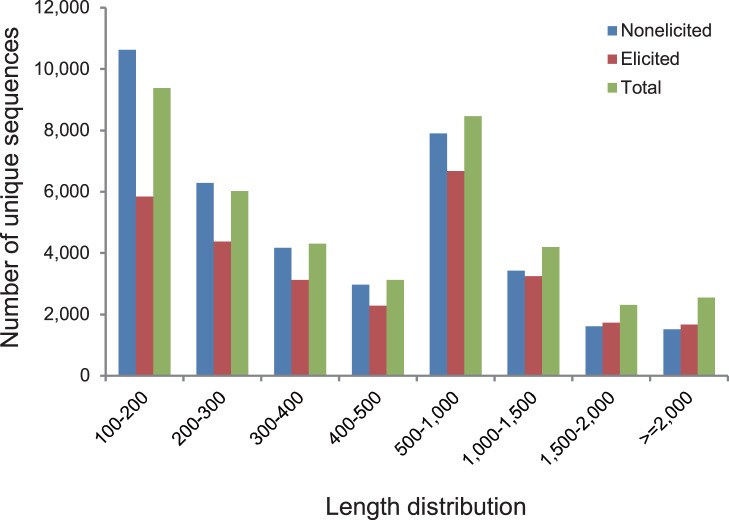
Length distribution of contigs from the nonelicited and MeJA elicited cultures, and the integrated data from both cultures (Total) assembled by using ABySS and CAP3. The y-axis indicates the number of unique sequences with certain length. The x-axis indicates the length range of unique sequences.

The assembly was independently carried out for each sample using ABySS [14]. From the nonelicited culture, a total of 38,506 contigs with length not less than 100 nt were generated; the N50 size and the mean size are 976 and 581 nt, respectively. Figure 1 shows the length distribution of contigs ranging from 100 nt to more than 2000 nt. From the elicited culture, 28,959 contigs have the N50 size of 1,142 nt and the mean size of 689 nt. We obtained a total of 40,348 unique sequences by further assembly from the two datasets with the N50 size of 1,193 nt and the mean size of 684 nt, of which 1368 probably are complete cDNAs.

### Functional Prediction of Unique Sequences by BLAST Search Against GenBank nr Protein Databases

To gain a preliminary insight into the functions of the unique sequences obtained, we performed BLASTX search against GenBank non-redundant (nr) using E-value 10^−5^ as a cutoff value. 21,652 out of 40,348 unique sequences (53.7%) were detected to have homologs. We found 35 and 74 unique sequences with the relatively high abundance of more than 1000 transcripts per million (relative abundance) in the nonelicited and elicited cells, respectively (Table S1), among which ribosomal protein S2 and gibberellin 3-beta-dioxygenase 4-like protein are highly expressed in both datasets. Compared with the nonelicited culture, the elicited culture contains unique sequences encoding the enzymes in pathogenesis and ethylene biosynthesis such as ACC oxidase. These abundantly expressed genes in cultured cells are distinct from those in needles, which include genes encoding ribulose bisphosphate carboxylase (RuBisCO) and other proteins that involved in photosynthesis [18]. Interestingly, we note multiple sequences with unknown annotation. After domain search against PROSITE database (http://prosite.expasy.org), only one (Contig29843) was found containing putative domains. It would be intriguing to investigate what functions other sequences perform, given the high levels that these genes possess.

### COG and GO Classification and KEGG Pathway Enrichment of Unique Sequences

**Figure 2:**
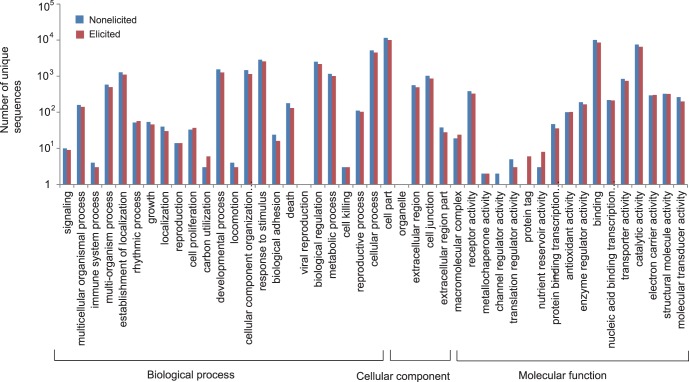
Histogram presentation of clusters of orthologous groups (COG) classification in nonelicited and MeJA elicited culture.

To facilitate the functional classification of the unique sequences, we employed their homologs’ functional classification information in COG database, which emphasizes a classification of genes in an evolutionary view using complete genome sequences [21]. According to COG database, all the unique sequences were classified into 25 different functional classes, which were represented by A to Z (Figure 2). About 19% and 23% unique sequences from the non-elicited cells and the elicited cells respectively were found homologs in COG database. The unique sequence frequency in each functional class varies from 0 to 1,886 in nonelicited cells and from 0 to 1,842 in the elicited cells. Compared with the nonelicited cells, the elicited cells showed more unique sequences in RNA processing and modification (Figure 2A, 5%), amino acid transport and metabolism (Figure 2E, 3%), and secondary metabolite biosynthesis, transport and catabolism (Figure 2Q, 9%) but have less expressed genes in other functional classes (from 1% to 24%).

**Figure 3:**
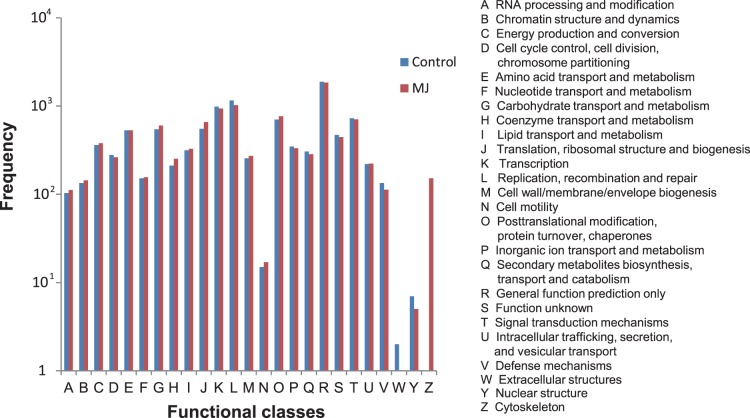
Histogram of Gene ontology (GO) classification of unique sequences in nonelicited and MeJA elicited cells. Results are categorized into three GO ontologies, biological process, cellular component, and molecular function. The classes without any unique sequences both in the nonelicited and elicited cells were removed from the figure.

After obtaining putative gene functions assigned by homology searches, we predicted the biological processes (26% and 30% unique sequences in non-elicited and elicited cells), cellular components (27% and 41% unique sequences in non-elicited and elicited cells) and molecular functions (30% and 46% unique sequences in non-elicited and elicited cells) that the proteins belong to, by association with GO information using the obtained annotation from GenBank nr database (Figure 3). The results showed that most proteins encoded by these unique sequences take part in the biological processes including response to stimulus, biological regulation, developmental process and metabolic process. As for the cellular component domain, a majority of products of unique sequences lie in cell part, cell junction, and extracellular region. For the molecular function domains, genes involved in binding and catalytic activity are dominant. Notably, almost all the classes have more unique sequences in the nonelicited cells than in the elicited cells (Figure 3).

To understand the metabolism pathways in nonelicited and elicited *T. × media* cells, we performed BLASTX search against KEGG database to map the unique sequences to canonical pathways. A total of 2,739 and 2,605 unique sequences in the elicited (9%) and non-elicited (7%) cells refer to 137 and 135 KEGG pathways, respectively (Table S2). Interestingly, the largest unique pathways in the two datasets include spliceosome (5%), ribosome (4%), protein processing in endoplasmic reticulum (3%), and purine metabolism (3%) (Table S2). Compared with the nonelicited cells, the elicited cells have slightly more unique sequences in the pathways including glycolysis/gluconeogenesis, DNA replication, mismatch repair, diterpenoid biosynthesis, etc. (Table S2).

### Comparison of the Expression Levels of Genes between Nonelicited and Elicited *T. × media* Cells

To investigate the response of elicitation with MeJA in *T. × media* cultured cells, we estimated the expression levels of each unique sequence in the two cultures by using RSEM software, which can quantify transcript abundance from RNA-seq data without a reference genome [15], and then performed statistical analysis implemented in EdgeR [16] using the mapped read numbers for each unique sequences calculated by RSEM. About 17% unique sequences (6,748) showed at least two fold changes (adjusted p-value ≤0.05) (Table S3). We found 282 unique sequences with no less than 4 fold changes (adjusted p-value ≤0.05), which are from 93 pathways (Table S4). As some unique genes are involved in more than one pathway, the total number of unique sequences in the 93 pathways reached to 708.

We investigated the expression changes in the view of pathways by calculating the genes with four fold changes. Among the 93 pathways, 26 showed increased transcript abundance, 17 of them displayed decreased transcript abundance, and other pathways had unique sequences with either increased or decreased in transcript abundance. Pathways that showed increased transcript abundance with at least two unique sequences include diterpenoid biosynthesis and terpenoid backbone biosynthesis. Interestingly, two pathways, biosynthesis of terpenoids and biosynthesis of alkaloids derived from terpenoid and polyketide, have at least 14 unique sequences with distinct changes and all but one had increased transcript abundance. Pathways which possessed decreased transcript abundance include for example cell cycle, base excision repair, and citrate cycle (TCA cycle). In addition, all the unique sequences in the pathways of DNA replication, mismatch repair, purine metabolism, homologous recombination, nucleotide excision repair had lower transcript abundance except for one unique sequence (Table S4).

### MeJA Responsive Genes Involved in the Regulation of Gene Expression

We examined the genes that regulate gene expression, including transcription factors and genes involved in DNA and histone modification. A total 411 unique sequences were identified as transcription factors, including multiple members from bZIP and bHLH transcription factor classes (Table S5). Among them, 46 unique sequences showed at least 4 fold changes (adjusted p-value <0.05). The expression levels of 21 out of the 46 transcription factors showed higher transcript abundance, including members of bHLH, bZIP, TRY, GRAS, WRKY, PLATZ, MYB, and AP2/ERF domain-containing transcription factors; the remaining 25 transcription factors displayed lower transcript abundance, including GRAS, WRKY, E2F, PLATZ, ICE1, DP, TRY, GT-2, and ethylene-responsive transcription factors (Table S5).

We also found 23 unique sequences encoding DNA methyltransferase, 13 encoding methyl-CpG-binding domain-containing proteins, 44 of histone methyltransferase, 16 of histone demethylase, 12 of histone acetyltransferase, 27 of histone deacetylase, and 2 for histone ubiquitination (Table S6). Interestingly, the expression levels of 5 DNA methyltransferases, 2 histone methyltransferases, and 2 histone deacetylases possessed lower transcript abundance. However, 1 histone demethylase and 2 histone deacetylases, displayed at least 3 times increased transcript abundance (adjusted p-value≤0.05).

### The Response of Genes in the Biosynthesis of Jasmonic Acid and Ethylene

Because the biosynthesis of JA is potentially important to the signal transduction of MeJA, we investigated the expression changes of genes in the two pathways of JA synthesis. Two of the seven genes in the biosynthesis of jasmonic acid, lipoxygenase (*LOX*) and allene oxide synthase (*AOS*), showed increased transcript abundance (Table S7). Similarly, methyl jasmonate esterase (*MJE*, Contig11373), catalyzing the conversion of MeJA to JA, also showed 5 fold increases in transcript abundance (Table S7). Two of the three genes involved in ethylene biosynthesis [22] were identified in our study, ACC synthase and ACC oxidase (Table S7). However, only ACC oxidase displayed distinct changes in expression levels. The expression levels of one unique sequence (Contig16235) which possessed 95% identity with the reported ACC oxidase in *T. cuspidata* [20] showed 4 fold increase in transcript abundance.

### Genes in the Main Chain of Terpenoid Backbone Biosynthesis and Paclitaxel Biosynthesis, Transport and Degradation and their Transcript Changes

Because the paclitaxel biosynthesis pathway is incomplete in the KEGG database, to find the genes responsible for the biosynthesis of the main chain of terpenoid backbone, we manually identified all genes involved in these two pathways by reciprocal BLAST search against the transcriptome using previous reported enzymes as queries (Table S8). The main chain of terpenoid backbone biosynthesis includes the mevalonate (MVA) pathway and the 2-C-methyl-D-erythritol 4-phosphate (MEP) pathway and requires 17 genes in total. Thirty-six unique sequences were obtained by manual BLAST search and all the 17 genes in the main chain of terpenoid backbone biosynthesis were found (Table 1 and Table S9). Interestingly, all the 10 unique sequences from eight genes had increases in transcript abundance. The largest increase in transcript abundance observed in the pathway were from 1-deoxy-D-xylulose-10-phosphate synthase (*DXS*, 35 and 15 fold), geranylgeranyl diphosphate synthase (*GGPPS*, 17 fold) (Table 1). Two unique sequences of 4-hydroxy-3-methylbut-2-enyl diphosphate reductase (*IDS*) show similar transcript abundance changes, 7 and 5 times, respectively, and as they encode different regions of IDS protein it is likely that they are from the same *IDS* gene. Other genes showing increased transcript abundance in the pathway include 4-hydroxy-3-methylbut-2-enyl diphosphate synthase (*HDS*), 4-diphosphocytidyl-2-C-methyl-D-erythritol kinase (*CMK*), 2-C-methyl-D-erythritol 2,4-cyclodiphosphate synthase (*MCS*), 1-deoxy-D-xylulose-5-phosphate reductoisomerase (*DXR*), and acetyl-CoA acetyltransferase (*AACT*) (Table1 and Table S9).

**Table 1:** Genes in main chain of terpenoid backbone biosynthesis and paclitaxel biosynthesis with changes in transcript abundance.

Sequence_ID	Annotation	Abbreviation	Subject IDs	Identities	Fold change
Terpenoid backbone biosynthesis					
237932	1-deoxy-D-xylulose-10-phosphate synthase	DXS	AAS89342.1	99%	36.1
Contig30899	Geranylgeranyl diphosphate synthase	GGPPS	AAD16018.1	99%	17.9
Contig29438	1-deoxy-D-xylulose-9-phosphate synthase	DXS	AAS89342.1	98%	15.8
Contig22315	4-hydroxy-3-methylbut-2-enyl diphosphate reductase	IDS	ABU44490.1	99%	6.8
Contig33460	1-deoxy-D-xylulose-5-phosphate reductoisomerase	DXR	AAT47184.1	99%	5.8
Contig28325	2-C-methyl-D-erythritol 2,4-cyclodiphosphate synthase	MCS	ABB88956.1	94%	5.3
Contig25640	4-hydroxy-3-methylbut-3-enyl diphosphate reductase	IDS	ABU44490.1	99%	5.2
Contig26664	4-diphosphocytidyl-2-C-methyl-D-erythritol kinase	CMK	AAZ80384.1	71%	4.9
Contig33194	4-hydroxy-3-methylbut-2-enyl diphosphate synthase	HDS	ABB78087.1	87%	4.8
Contig33656	acetyl-CoA acetyltransferase	AACT	NP_851150.1	81%	3.7
Paclitaxel biosynthesis					
Contig33713	taxane 2α-O-benzoyltransferase	TBT	Q9FPW3.1	96%	n.d.
Contig29447	taxane 2α-O-benzoyltransferase	TBT	Q9FPW3.1	93%	47.4
Contig28471	3′-N-debenzoyl-2′-deoxytaxol N-benzoyltransferase	DBTNBT	Q8LL69.1	99%	32.1
Contig32749	Taxane 7-beta hydroxylase	T7OH	ACR78247.1	99%	31.0
203604	3′-N-debenzoyl-2′-deoxytaxol N-benzoyltransferase	DBTNBT	Q8LL69.1	97%	25.8
Contig19765	Taxane 5-alpha hydroxylase	T5OH	AAU93341.1	100%	23.6
Contig30864	taxadien-5α-ol-O-acetyl transferase	TAT	AAU89980.1	93%	22.2
Contig28796	taxadien-5α-ol-O-acetyl transferase	TAT	AAU89980.1	99%	22.1
Contig20518	10-deacetylbaccatin III-10-O-acetyltransferase	DBAT	ABW84241.1	100%	19.2
Contig24572	3′-N-debenzoyl-2′-deoxytaxol N-benzoyltransferase	DBTNBT	Q8LL69.1	99%	17.8
247064	taxa-4(5),11(12)-diene synthase	TS	AAZ41362.1	100%	17.2
Contig17866	Taxane 5-alpha hydroxylase	T5OH	AAU93341.1	100%	16.0
240648	baccatin III 3-animo-3-phenylpropanoyltransferase	BAPT	ACN62085.1	99%	14.9
Contig26380	Taxane 5-alpha hydroxylase	T5OH	Q6WG30.2	100%	12.2
Contig22452	10-deacetylbaccatin III-10-O-acetyltransferase	DBAT	ABW84241.1	99%	12.2
Contig20363	taxadien-5α-ol-O-acetyl transferase	TAT	AAS49031.1	100%	11.5
Contig21313	3′-N-debenzoyl-2′-deoxytaxol N-benzoyltransferase	DBTNBT	AAT73199.1	98%	10.3
Contig26054	taxa-4(5),11(12)-diene synthase	TS	AAR13861.1	96%	10.3
Contig17837	taxadien-5α-ol-O-acetyl transferase	TAT	AAS49031.1	100%	10.0
Contig27532	taxadien-5α-ol-O-acetyl transferase	TAT	Q8S9G6.1	99%	8.5
Contig17061	3′-N-debenzoyl-2′-deoxytaxol N-benzoyltransferase	DBTNBT	AAT73199.1	99%	7.8
Contig18229	taxa-4(5),11(12)-diene synthase	TS	AAZ41362.1	99%	7.6
Contig30915	Taxane 10-beta hydroxylase	T10OH	Q9AXM6.1	99%	6.4
Contig33190	baccatin III 3-animo-3-phenylpropanoyltransferase	BAPT	ACN62085.1	99%	6.3

For the genes in paclitaxel biosynthesis, only genes with more than 90% identity with reported genes are shown. “n.d.” indicates that the estimated relative abundance is zero in the nonelicited culture.

The paclitaxel biosynthesis pathway is still not fully elucidated and only 12 genes are confirmed (Figure S2). A manual BLAST search obtained 130 unique sequences from 11 of 12 genes, with only phenylalanine aminomutase (*PAM*) missing (Table S10). We searched the assembly results using an alternative assembly program SoapDenovo [23] and found 5 and 8 short *PAM* fragments in the nonelicited and elicited cultures, respectively. Among the 11 reported genes, *T13OH* shows the highest 69% identity with one unique sequence and the other ten genes display at least 90% identities to multiple unique sequences.

Of the 130 unique sequences associated with the paclitaxel biosynthesis pathway, 108 showed increased transcript abundance. Nine of 108 showed extremely low transcript levels in the nonelicited cells and therefore their expression changes could not be determined, which encode taxadien-5α-ol-O-acetyl transferase (*TAT*), taxane 2α-O-benzoyltransferase (*TBT*), taxane 10-beta hydroxylase (*T10OH*), 3′-N-debenzoyl-2′-deoxytaxol N-benzoyltransferase (*DBTNBT*), and taxane 5-alpha hydroxylase (*T5OH*). The largest expression changes in the remaining genes were 7 fold transcript abundance in baccatin III 3-animo-3-phenylpropanoyltransferase (*BAPT*) and 10-deacetylbaccatin III-10-O-acetyltransferase (*DBAT*), 6 fold in taxane 5-alpha hydroxylase (*T5OH*), taxane 7-beta hydroxylase (*T7OH*), and taxa-4(5), 11(12)-diene synthase (*TS*). The three unique sequences with lower transcript levels encode taxadien-5α-ol-O-acetyl transferase (*TAT*) and taxane 5-alpha hydroxylase (*T5OH*) (Table 1 and Table S10).

We also obtained some candidates of the unknown enzymes in paclitaxel biosynthesis among the unique sequences that show increases in transcript accumulation (Table S11 and Figure S2). By searching the annotations proposed in previous studies [24], [25], we found 2 unique sequences for epoxidase, 6 for oxomutase, 9 for CoA ligase, and 1 for oxidase. By blast search, we obtained 27 unique sequences encoding P450s that may function as T1OH, T9OH and T2’OH. Most of these candidates showed more than four-fold changes after elicitation of MeJA.

Some transporters, such as ABC transporters, are thought to be involved in the transport of paclitaxel [26], and 47 unique sequences encoding such transporters were found with distinct changes in expression levels. Among them were one unique sequence with increased transcript abundance and six unique sequences with lower transcript abundance (Table S12). In addition, 60 genes potentially associated with paclitaxel degradation, including diverse esterase and hydrolase show distinct changes (Table S13). However, although 10 unique sequences encoding multidrug and toxic compound extrusion transporters (MATE) were identified, none show significant changes in transcript abundance.

### Comparison of MeJA Altered Transcript Levels between *T. × media, T. chinensis*, and *T.cuspidata*

By using broad gene functional descriptions our gene expression data was compared to other published studies investigating MeJA gene expressions changes in other *Taxus* species. Between *T. × media* and *T. chinensis* we identified seven genes that may function as transcription factors that were identified as possessing abundantly increased transcript levels in our data but not in the results of Li et. al. [19]; including GATA domain class transcription factor, PLATZ transcription factor, BIM1-like, E2FB-like, FER-Like iron deficiency-induced, HY5-like, and TT8 transcription factors. For genes involved in paclitaxel biosynthesis, only 9 of 12 genes with increased transcript levels after MeJA elicitation were also identified as similarly up-regulated by Li et al. [19] (*T2OH*, *T13OH*, and *TBT* were not identified as up regulated).

Only about 22.7% genes with distinct expression changes in *T. cuspidata* were found in our study and 70 of these genes showed consistent changes to the elicitation of MeJA in the two cell lines with different sampling times (Table S14). Twenty-two unique sequences with lower transcript level changes in *T. × media* also had lower transcript levels in *T. cuspidata*. Among the genes with lower transcript levels in both datasets, HD2 type histone deacetylase is known to be involved in jasmonic acid and ethylene signaling in response to pathogens [27]. Similarly, 26 unique sequences with increased transcript changes in *T. × media* were also up-regulated in *T. cuspidata*, which contained 29 genes. These include *TBT* in paclitaxel biosynthesis [28], 1-aminocyclopropane-1-carboxylate oxidase (*ACO*) in ethylene biosynthesis [22], 12-oxophytodienoate reductase possible in jasmonate biosynthesis [29], cationic peroxidase in pathogen incompatible interaction [30], Chalcone synthases involve in phenylpropanoid biosynthesis which is inducible by pathogens [31], and taxane 14b-hydroxylase participating in the bifurcation in paclitaxel biosynthesis [32]. Notably several genes between the different studies were identified as having opposite transcript changes in response to elicitation with MeJA: 15 genes (include one of two ACC oxidases) in *T. × media* with lower transcript levels were up-regulated in *T. cuspidata*,while 7 genes(e.g., dihydroflavonol-4-reductase in anthocyanin biosynthesis [33]) in *T. × media* with increased transcript levels were identified as down-regulated in *T. cuspidata*.

### Identification of Potential mRNA Targets by miRNAs

Specific miRNAs have been implicated in the regulation of gene expression of *T. chinensis* cells elicited with MeJA [33]. To investigate the potential role of miRNAs in the synthesis of paclitaxel in *T. × media* cells, all unique sequences that were potential targets for MeJA regulated miRNAs were identified [33]. In total 200 unique sequences with assigned functions by homology searches, 106 showed changes in transcript abundance (Table S15). Interestingly, increased transcripts encoding the transcription repressor MYB4, myb-related protein 308, ethylene-responsive transcription factor ERF113, and acyltransferase, are all predicted to be the targets of miR-858, miR837, miR172, miR408, miR169, miR397b, miR172e, and miR396a that have lower transcript numbers in *T. chinensis* following the elicitation of MeJA [33], whereas sequences with lower transcript accumulation encoding alpha-expansin 15 are potential targets of miR164ab and miR164c, which have higher transcript numbers in *T. chinensis* after the elicitation of MeJA. These results indicate that these predicted targets may be valid targets of miRNAs (Table S15).

### Validation of Differentially Expressed Genes with qRT-PCR Analysis

**Figure 4:**
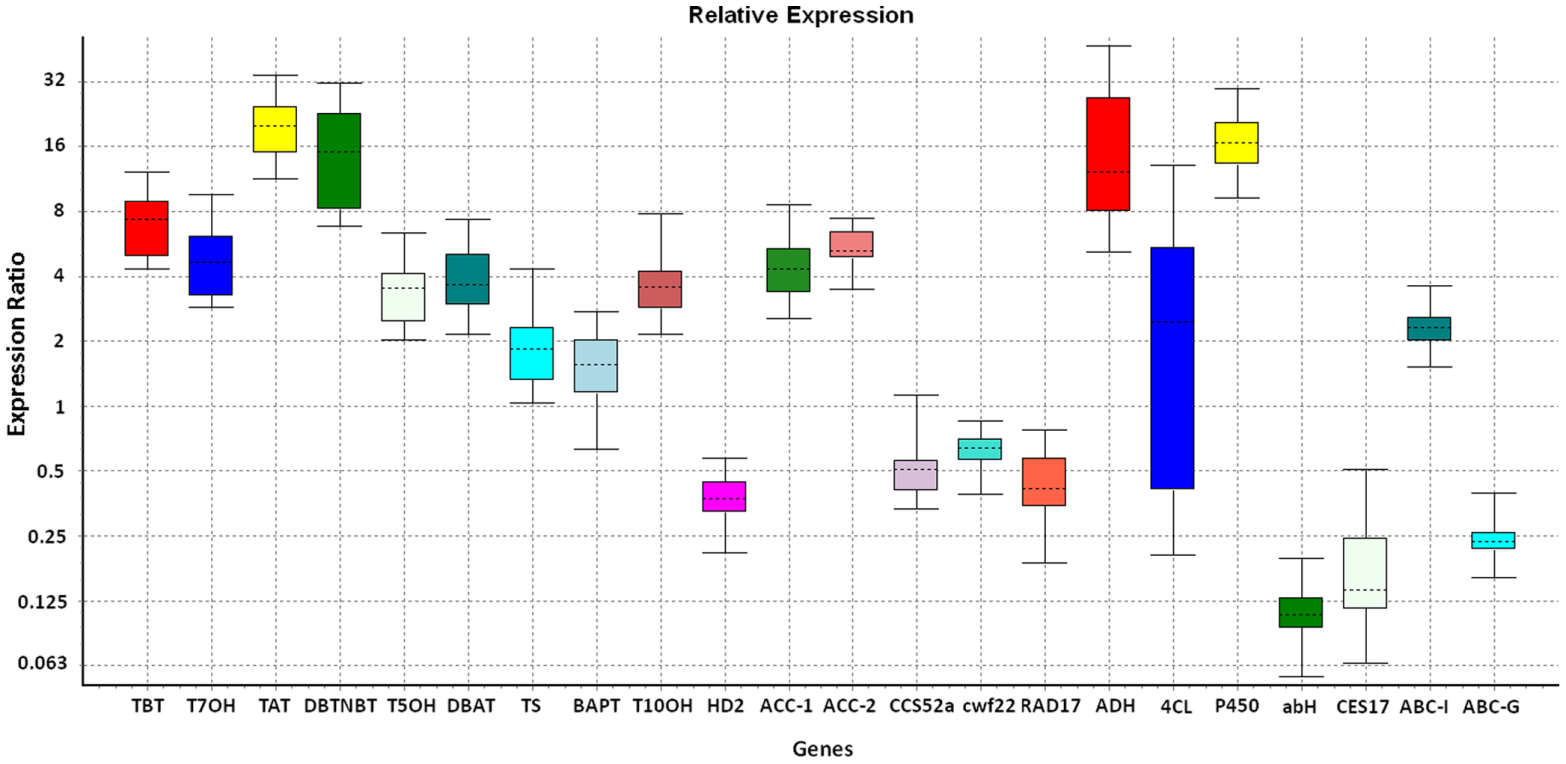
Expression analysis of 22 representative genes in nonelicited and elicited cells by qRT-PCR. Y-axis indicates the relative expression distribution of a ratio of elicited cells versus nonelicited cells with *actin* as the reference gene. X-axis displays 22 selected genes, whose full names were given in Table 2. The boxes indicate 50% of observations near the median represented by the dotted line, and the whiskers show the outer 50% of observations.

To validate the RNA-seq gene expression results, we selected 22 representative genes encoding 7 groups of enzyme types (Table 2) and designed specific primers for quantitative real-time PCR (Table S16). Consistent with RNA-seq analysis, the relative expression pattern of 20 genes out of 22 tested genes were identified as being significantly up-regulated or down-regulated in the MeJA induced cell cultures as compared to the control (Table 2, Figure 4). Contig787 (cell cycle switch protein CCS52a) has different expression pattern with the RNA-seq analysis. The expression change of 133463 (AMP dependent CoA ligase) is not significant in qRT-PCR assays, while the RNA-seq analysis presumed that it should be up-regulated by MeJA treatment. It is notable that all 9 genes involved in paclitaxel biosynthesis are up-regulated, and 2 candidate genes involved taxol degradation are down-regulated following MeJA treatment with both qRT-PCR and RNA-seq analysis.

**Table 2:** Comparison of the expression change of the 22 selected candidate genes with RNA-seq and qRT-PCR analysis.

Putative Function	IDs	Putative Function	Fold change	
			RNA-seq	qRT-PCR
	Contig31917	taxane 2α-O-benzoyltransferase, TBT	17.1	6.9
	Contig32749	taxane 7β-hydroxylase, T7OH	48.5	4.7
	Contig30864	taxadien-5α-ol-O-acetyl transferase, TAT	34.3	19.2
Paclitaxel biosynthesis	Contig28471	3′-N-debenzoyl-2′-deoxytaxol N-benzoyltransferase, DBTNBT	52.0	14.2
	Contig19765	cytochrome P450 taxadiene 5α-hydroxylase, T5OH	29.9	3.3
	Contig20518	10-deacetylbaccatin III-10-O-acetyl transferase, DBAT	21.1	3.9
	247064	taxa-4(5),11(12)-diene synthase, TS	17.1	1.8
	240648	baccatin III 3-animo-3-phenylpropanoyltransferas, BAPT	14.9	1.5
	Contig30915	taxane 10β-hydroxylase, T10OH	5.7	3.6
HD2 type histone deacetylase	120154	HD2 type histone deacetylase, HD2	0.8	0.4
ACC oxidase	Contig16235	ACC oxidase, ACC-1	4.6	4.4
	235821	1-aminocyclopropane-1-carboxylate oxidase, ACC-2	24.3	5.4
	Contig787^#^	cell cycle switch protein CCS52a, CCS52a	4.9	0.5
Cell cycle	Contig18564	cell cycle control protein cwf22, cwf22	0.4	0.6
	114663	cell cycle checkpoint protein RAD17-like, RAD17	0.5	0.4
Candidates in the	Contig24302	short chain alcohol dehydrogenase, ADH	14.9	14.8
unknown steps of	133463^#^	AMP dependent CoA ligase, 4CL	26.0	1.7^*^
paclitaxel biosynthesis	Contig24891	cytochrome P450 716B2, P450	12.1	17.0
Taxol degradation	Contig17145	alpha/beta hydrolase fold-containing protein, abH	0.1	0.1
	84500	carboxylesterase 17, CES17	0.1	0.2
Taxol transport	Contig20317	ABC transporter I family member 6, chloroplastic, ABC-I	2.8	2.3
	241293	ABC transporter G family member 3, ABC-G	n.d.	0.2

“^#^” indicate different expression models in RNA-seq analysis and qRT-PCR assays. “n.d.” indicates that the expression ratio of the gene in elicited cells to that in nonelicited cells approaches to zero. “^*^” indicates that the p-value is 0.447.

## Discussion

### Transcriptome Re-programming of *T. × media* Cells After Elicitation with MeJA

Plant cell culture provides an alternative source to produce secondary metabolites including pharmaceuticals, nutraceuticals, and food additives [34], [35]. Different cell lines of *Taxus* were established but the low yield of paclitaxel has been hindering the commercial application. Phytohormone MeJA not only regulates normal developmental processing but also induces defensive responses to herbivores and pathogens by accumulating secondary metabolites [36]. In an effort towards establishing high paclitaxel producing cell cultures, the plant hormone MeJA was used to elicit the accumulation of paclitaxel. The highest production reaches to 36.0 mg/L after 7 days elicitation with 200 µM MeJA [9], which represents the steady state of paclitaxel biosynthesis and accumulation. As only one biological replicate was sequenced from elicited and nonelicited cells, a limitation of our analysis is that we cannot determine the biological variation of genes we identify as being MeJA responsive. Our list includes false positives as we find the results of 2 out of 22 tested genes are not consistent with the qRT-PCR analysis. Although good consistent changes were obtained for most candidate genes, it is still required to conduct independent validations to accurately measure the expression levels of genes of interest.

By *de novo* assembly, we obtained 38,506 and 28,959 contigs from the nonelicited and elicited cultures, respectively. Total 40,348 unique sequences were acquired by further assembly from these two datasets. Analysis on the expression levels revealed that the elicitation of MeJA induced the transcriptome re-programming in *T. × media* cells. We tracked the genes in the signaling network that MeJA triggered as an elicitor [37], [38]. A number of genes were found with transcript abundance changed, which included transcription factors, DNA and histone modification proteins, and the down-stream target genes involved in the biosynthesis of the main chain of terpenoid backbone, paclitaxel, jasmonic acid and ethylene. Among the transcription factors that were identified, Apetala2/ethylene response factor (AP2/ERF)-domain, R2R3 MYB, and MYC transcription factors are MeJA-modulated regulators of secondary metabolite biosynthesis [39], [40], [41]. HY5, a bZIP transcription factor, was recently shown involving in MeJA signaling pathway by interaction with MYC2 transcription factor [42].

Interestingly, we found some other bHLH and bZIP transcription factors, which play central roles in phytochrome signal transduction [43] and are involved in light and stress signaling processes [44], respectively. This implies that supplementation of MeJA may change light signal transduction network. However, the changes of expression levels in the two classes are inconsistent, with some members with increased transcript abundance while others have lower abundance. Considering that bZIP and bHLH transcription factors are large families, different members of these classes probably take part in functionally diverse processes and therefore show diverse changes in expression levels. Similarly, the complex expression changes of WRKY transcription factors following the elicitation of MeJA are consistent with their known diverse functions in biotic and abiotic stresses [45].

### Key Regulatory Genes Involved Paclitaxel Biosynthesis, Transport, and Degradation

We found all 17 genes involved in the main chain of terpenoid backbone biosynthesis and 12 genes in paclitaxel biosynthesis, which include 36 and 167 unique sequences in the two biosynthesis pathways respectively. They provide a valuable start point to discovering key regulatory points in the synthesis of paclitaxel. Seven genes in the main chain of terpenoid backbone biosynthesis show changes in transcript abundance. The increased transcript abundance of *GGPPS* (18 times) was also observed by northern blot analysis in *T. × media* cells cultured on solid medium [46]. Random sequencing of cDNA clones derived from a MeJA elicited cell culture implicated *DXR* as being potentially MeJA inducible [47], and our data confirm this, as its transcript levels were 6 times higher.

Eleven of the 12 genes in paclitaxel biosynthesis displayed increased transcript abundance, 6 of which were reported by Lenka et al [20] and 8 by Li et al. [19]. The genes with increased transcript abundance in our data but absent from Li et al.’s data [19] imply that they are specifically increased in the steady state of paclitaxel production but not in the early state of MeJA elicitation. Intriguingly, we also found multiple candidates in the unknown steps of paclitaxel biosynthesis. Further experimental evidence is needed to confirm whether their putative function in paclitaxel biosynthesis. Xylosyltransferase can act as a shunt of paclitaxel biosynthesis by transferring a xylosyl group to the intermediate metabolites of paclitaxel biosynthesis including 10-deacetylpaclitaxel and 10-deacetyl cephalomannine and therefore decrease the intermediates fluxed to paclitaxel. We found 14 unique sequences annotated as xylosyltransferase but none showed changes in transcript abundance to the elicitation of MeJA, implying that the shunt to other chemical compounds is unlikely during elicitation of MeJA.

The transport of paclitaxel is bidirectional as extracellular paclitaxel can be absorbed into the cells and intracellular paclitaxel may be secreted into the extracellular medium [48]. Previous studies implied the presence of a specific transport pathway for the paclitaxel, although the identity of the transporters was unknown [26], [48]. ABC transporters have been previously proposed to be responsible for paclitaxel transport [26] and our data showed that 6 of 45 unique sequences encoding s showed lower transcript abundance. We also found one ABC transporter with higher transcript abundance and the significant expression change was also validated by qRT-PCR assay, which supports their association with the efflux/influx of paclitaxel. Further experiments on these candidates may help to identify the proteins involved in paclitaxel transport and aid in the bioengineering of paclitaxel extraction [48].

After synthesis, a portion of the paclitaxel synthesized is transported into the vacuole for storage [49]. Multidrug and toxic compound extrusion transporter (MATE) has been reported to involve in the vacuolar transport of some secondary metabolites, including alkaloids [50] and flavonoids [51], [52]. We found 10 unique MATE sequences, none of which showed distinct transcript changes. This implies that MATE may not be involved in the transport paclitaxel into the vacuole. Among those esterase and hydrolase genes that potentially associated with paclitaxel degradation, the genes showing lower transcript profiles, such as alpha/beta hydrolase fold-containing protein whose significant decrease in expression level was validated by qRT-PCR assay, are potential targets for enhancing paclitaxel production in *Taxus* cell culture with gene knock-out or knock-down strategy.

### The Response of Genes in Jasmonic Acid and Ethylene Biosynthesis

MeJA cannot activate gene expression until it is converted to jasmonic acid (JA) and further to bioactive JA-Ile derivative [36]. Besides derived from MeJA, JA can also *de novo* biosynthesized from linolenic acid in chloroplast. The increased transcript abundance of genes in the biosynthesis of JA is consistent with the known self-activation of jasmonic acid biosynthesis from previous studies [53]. The interaction between JA and ethylene signaling pathways affects accumulation of plant secondary metabolites [54] and ethylene, whose accumulation can also enhance the biosynthesis of paclitaxel in *T. cuspidata* cells together with MeJA [55]. Therefore it is interesting to investigate the gene expression changes of genes in ethylene biosynthesis. Of note, one unique sequences (Contig16235) encoding ACC oxidase with 4 times up-regulation was estimated to have 1,875 transcripts per million, which is one of the genes with highest transcript numbers. The increase of ACC oxidase implies higher accumulation of ethylene, which can also enhance the biosynthesis of paclitaxel in *T. cuspidata* cells together with MeJA [55].

### The Modulation Mechanism of MeJA on the Cell Growth

Paclitaxel is produced as a secondary metabolic product by the host plants to defend pathogens, such as oomycetes and wood decaying fungi [56]. Following the metabolic flux is shunt to various secondary metabolites, the nutrients used for the normal growth of plants or cells were affected and the plants or cells undergo retarded development. However, how such a resource reallocation occurs, and how MeJA mediates the crucial switch from growth to defense is still unclear. The growth repression by MeJA was found to be associated with reduced transcript levels of genes involved in cell cycle, especially the M-phase genes [57]. We found that two genes associated with the cell cycle, DNA-dependent protein kinase and minichromosome maintenance protein, showed four fold decreased transcript abundance, of which minichromosome maintenance protein reportedly displayed up-regulation changes in the fast growing cells [58]. Interestingly, besides the cell cycle progression, our results also revealed that the expression levels of the genes involved in DNA replicates, meiosis, and mismatch repair had decreased transcript abundance, which included DNA replication licensing factor MCM, proliferating cell nuclear antigen, beta-lactamase class C protein, DNA mismatch repair protein, and exonuclease. This implies that MeJA also modulates the growth and development of cells by interfering with the cell cycle, while they mobilize the resources to produce large amount of secondary metabolites for defense.

### Expression Change Comparison of Genes in the Early and Steady Elicitation of MeJA

Li et al. [19] investigated the global changes of *T. chinensis* transcriptome in the early MeJA elicitation events (16h after MeJA addition), and Lenka et al. [20] sampled the cells at 6 hours, 18 hours, and 5 day post-elicitation in *T. cuspidata*. Therefore, it is interesting to know the change profile of genes along with the treatment of MeJA. Unfortunately, since the original raw RNA-seq data is not available we could only identify genes in this study based on broad functional descriptions to compare transcript changes. Seven genes encoding transcription factors and three genes in paclitaxel biosynthesis were uniquely found with increased transcriptional abundance in our data. The genes with similar and distinct expression models in our data and in *T. cuspidata* potentially provides us a set of targets for the following metabolic engineering to directly increase paclitaxel production, such as *TBT* in paclitaxel biosynthesis [28] and the gene expression regulator HD2 type histone deacetylase in epigenetics [27]. Notably, taxane 14b-hydroxylase directs a side-route of paclitaxel pathway which can shunts paclitaxel production, and transcription inhibition to this gene can decrease the product of other compounds [59]. The unique sequence encoding taxane 14b-hydroxylase may provide a good target to indirectly increase paclitaxel production. Further research on these genes specifically their temporal response to MeJA elicitation would provide valuable information about the regulatory mechanism of paclitaxel biosynthesis.

### The Regulatory Roles of miRNAs in the Elicitation of Methyl Jasmonate

Qiu et al [33] identified 58 miRNAs from 25 families in *T. chinensis* cells with 14 miRNAs (7 families) showing MeJA altered expression. The lack of extensive sequence information on the *T. chinensis* genome precluded further identification of *Taxus* specific targets in that study. However because of the high conservation of miRNAs among closely related species in plants [60] and the stringent complementarity between plant miRNAs and their mRNA targets, we can use the mRNA sequences from *T. × media* in this study to identify potential mRNA targets. Two hundred potential targets with known functions were found (Table S15) and multiple pairs of miRNAs and predicted targets show complementary expression patterns (higher and lower transcript abundance), including transcription factors and other regulatory genes possibly involved in the biosynthesis of paclitaxel, such as MYB, ethylene-responsive transcription factors, acyltransferase, and geranylgeranyl transferase type I. Among these targets, Courdavault et al. once proposed that geranylgeranyl transferase type I is involved in early jasmonate signaling transduction pathway [61]. The complementary expression patterns of predicted targets with miRNAs provides further support for the notion that miRNA-mediated gene expression regulation may also involve in the signal network following the elicitation of MeJA, including the biosynthesis of paclitaxel. It may be possible to develop a miRNA-based method for improving paclitaxel yield in the future.

## Materials and Methods

### Plant Materials

As solid cell culture has simpler and more reproducible generation procedures, but possesses similar growth characteristics as suspension cell culture, solid medium was used to culture all material in this study. The cell line was created from leaf segments of *Taxus × media* on the B5 medium containing 100 mg/L myo-inositol, 1.25 mg/L nicotinic acid, 1.0 mg/L vitamin B1, 0.5 mg/L vitamin B6, 1.0 g/L lactalbumin hydrolysate, 0.1 mg/L 6-BA, 1.0 mg/L 2,4-D, 8.0 g/L agar and 20.0 g/L sucrose. Subculture was carried out every four weeks on 67-V solid medium supplemented with 2.0 mg/L IAA, 1.5 mg/L 2,4-D, 0.2 mg/L 6-BA, 100 mg/L myo-inositol, 8.0 g/L agar and 30 g/L sucrose. *T*. *× media* is a hybrid designation for crosses between English yew (*Taxus baccata*) and Japanese yew (*Taxus cuspidata*). These hybrids are noted for high palcitaxel content in their needles and shoots. The elicitor, methyl jasmonate (MeJA) was dissolved in an equal volume ethanol, filter-sterilized and then added to the media before solidification with the final concentration of 200 µM. For the control experiment, an equivalent amount of ethanol was added to the medium. The cells were harvested on 7 days after elicitation for total RNA isolation.

### Total RNA Isolation and cDNA Library Construction

The total RNAs were extracted using a modified CTAB method from Chang [62]. Briefly, 1g of fresh cells was ground in liquid nitrogen and the powder was mixed with 100 µL mercaptoethanol and 5 mL extraction solution preheated to 65°C. After a water bath at 65°C for 3 min, the mixtures were centrifuged for 10 min at 12,000 rpm at 4°C. The upper aqueous phase was extracted two times with equal volume of chloroform:isoamyl alcohol (24∶1). Then, 1/4 volume 10 M LiC1 was added to the supernatant. After placement overnight at −20°C, the precipitation was collected by centrifuging for 20 min at 12,000 rpm at 4°C and dissolved in 500 µL SSTE. The solution was further extracted with equal volume of phenol:chloroform:isoamyl alcohol (25∶24:1) and with 2.5 time volume of ethanol. The precipitate was dried and dissolved in water. The quantity and quality of total RNA were evaluated using a Nanodrop ND-1000 (Nanodrop technologies, Wilmington, DE, USA), gel electrophoresis and Agilent 2100 (Agilent Technologies, Palo Alto, Calif.). RNA samples showing good quality in RNA electrophoresis and RNA integrity number more than 8.4 in Agilent 2100 were stored at −80°C until sequencing. The cDNA libraries were constructed by using Illumina’s kit following the manufacturer’s protocol (TruSeq RNA Sample Preparation Kits v2, San Diego, USA).

### Sequencing and Data Analysis

The paired-end cDNA library sequencing was performed on an Illumina Solexa 1G Genome Analyzer. Image deconvolution and quality calculation were conducted by using the Illumina GA pipeline 1.3. Adaptor sequences, empty reads and low quality sequences were removed from the raw reads to produce high quality reads. The high quality reads were then assembled by using ABySS (version 3.8) [14]. In brief, all the high quality reads were firstly assembled into contigs using different k-mer values, then the assembly results from diverse k-mer values were assessed based on the total number of contigs with length no less than 100 nt, the N50 value, the median and mean lengths of contigs. The optimal assemblies were selected for the control and MeJA treatment datasets. For the convenience of comparison on gene expression levels, the contigs of the two dataset was assembled into one integrated dataset using CAP3 [63]. We call the resulting integrated sequence fragments as unique sequences in this study.

After removing all the unique sequences with ambiguous or unknown bases, the number of complete cDNAs was estimated as the following: the putative open reading frames of all unique sequences were predicted with OrfPredictor (http://proteomics.ysu.edu/tools/OrfPredictor.html), which were then used as queries to search against Swiss-Prot database with E-value set to 1e-5. The unique cDNAs were considered complete cDNAs if the alignment coverage of the top hit was more than 90%.

Unique sequences were used for BLAST search and annotation against NCBI non-redundant (nr) databases, cluster of orthologous groups of protein (COG) database, kyoto encyclopedia of genes and genomes (KEGG) database, and gene ontolog (GO) database with an cut-off E-value of 10^−5^, respectively. Functional annotations were performed by sequence similarity against nr database and the annotations of first sequence with highest sequence similarity and clear function annotation were endowed to the corresponding unique sequences. Both functional annotation by GO was analyzed against the GO database, and the pathways annotation was retrieved using the internal KEGG information of hits in GO database. The expression levels of unique sequences in each dataset were calculated by using RSEM software (version 1.1.13) [15]; the resulting mapped reads number for each unique sequences was feed to EdgeR (version 2.2.6) [16] to perform the significance analysis. The normalization factor was calculated using calcNormFactors, the common dispersion across the read numbers mapped to each unique sequences was estimated using estimateCommonDisp, the genewise exact test for difference in the means between the nonelicited and elicited cells of negative-binomially distributed counts was computed using exactTest. The unique sequences with no less than a twofold change in the transcript level and p-value ≤0.05 were considered as significantly differently expressed between the nonelicited cells and elicited cells.

### Homolog Search for Identifying Genes Involved in the Main Chain of Terpenoid Backbone Biosynthesis and Paclitaxel Biosynthesis

The genes involved in the main chain of terpenoid backbone biosynthesis and paclitaxel biosynthesis were manually identified by BLAST search against the assembled contigs. The queries were all from *Taxus* if available and the genes from *Arabidopsis thaliana* (TAIR, www.arabidopsis.org) were used if they are still unknown in *Taxus*. All the hits with E-value less than 10^−5^ in *Taxus* were then used as queries to search GenBank nr database again and were kept if their subjects also were annotated as the enzymes involved in the main chain of terpenoid backbone biosynthesis and paclitaxel biosynthesis.

### Comparison of Genes with Significant Expression Changes After Elicitation with MeJA between *T. × media* and *T. cuspidata*

The genes with significant changes in expression levels after elicitation with MeJA were compared between *T. × media* and *T. cuspidata* by blast search. All the unique sequences in our data showing two fold expression changes after elicitation with MeJA were used as queries to search against all the genes obtained by SSH [20]. All the subjects from *T. cuspidata* were kept if their identity values with *T. × media* and the alignment lengths are not less than 95% and 50 amino acids, respectively.

### Prediction of Potential Target mRNAs of miRNAs

The miRNAs found in *T. chinensis* [33] were used to identify the potential miRNA targets against all the assembly unique sequences with known functions by target-align (http://www.leonxie.com/targetAlign.php) [64], which was developed by ourselves. The sequences that satisfied the following parameters were considered as the potential targets of miRNAs: i) the total mismatched nucleotides and the contiguous mismatched nucleotides in the miRNA:mRNA duplexes were no more than four and two respectively; ii) the mismatched nucleotides from the first site to the ninth site in miRNAs do not exceed one; iii) no mismatch occurs on the tenth and eleventh sites; iv) the score for G:U match was set to 0.5.

### Real-time PCR Assays

Total RNA was extracted using Column Plant RNAout kit (TIANDZ, Beijing, China) from the *T. × media* MeJA elicited cells and nonelicited cells as those prepared for RNA-seq sequencing, according to the manufacturer's protocol. RNA was treated with DNase I (Takara, Dalian, China) to remove residual genomic DNA. The concentration of the isolated RNA and the 260/280-absorbance ratio was measured in triplicates with Nanodrop ND-8000 (Thermo, USA). The quality of RNA samples was confirmed by electrophoresis on a 1.2% agarose. Total RNA was reverse-transcribed to cDNA using PrimeScript RT Master Mix (Perfect Real Time) (Takara, Dalian, China) in a total volume of 20 µl containing 1 µg of total RNA. The reaction was carried out at 37°C for 15 min and 85°C for 5 s. Forward (Fwd) and reverse (Rev) primers were designed using Primer3 [65]. Table S16 shows the primers for the selected genes and the reference gene *actin*. The real-time PCR assays were conducted in an optional 96-well plate with ABI7500 system (ABI, USA) and a commercial SRBR-Green master mix kit (Takara, Dalian, China). The reactions were performed with 1 µl cDNA in 20 µl reaction mix containing 10 µl 2 × SYBR Premix Ex Taq and 1 µl primers. The conditions were as follows: initial holding at 95°C for 30 s, followed by a two-step program of 95°C for 5 s and 55°C for 34 s for 40 cycles. Two independent biological replicates, three technical replicates for each PCR reaction were performed. Reverse transcriptase negative controls and “no template controls” (without cDNA in PCR) were included. Data analysis was performed with REST 2009 Software [66]. Statistical difference are significant when p<0.05.

## Supporting Information

Figure S1 ***Taxus × media*** **cells cultured on solid medium.**
(DOC)

Figure S2 **Flow chart of paclitaxel biosynthetic pathway in** ***T. × media*** **.**
(DOC)

Table S1 **Unique sequences with over 1000 transcripts/million in nonelicited and elicited cultures.**
(XLS)

Table S2 **Comparison of unique sequences in KEGG pathways between nonelicited and elicited cultures.**
(XLS)

Table S3 **Unique sequences with no less than two-fold changes in transcript levels (adjusted p-value ≤0.05) following the elicitation of MeJA.**
(XLS)

Table S4 **KEGG pathway analysis of unique sequences with distinct changes in expression levels following MeJA elicitation.**
(XLS)

Table S5 **Transcription factors and expression levels comparison between nonelicited and elicited cultures.**
(XLS)

Table S6 **Genes involved in DNA and histone modification and expression level comparison between nonelicited and elicited cultures.**
(XLS)

Table S7 **Genes involved in biosynthesis of jasmonic acid and ethylene and expression level comparison between nonelicited and elicited cultures.**
(XLS)

Table S8 **Queries used in the BLAST search to identify genes in the main chain of terpenoid backbone biosynthesis and paclitaxel biosynthesis.**
(DOC)

Table S9 **Genes involved in main chain of terpenoid backbone biosynthesis and expression level comparison between nonelicited and elicited cultures.**
(XLS)

Table S10 **Genes involved in paclitaxel biosynthesis and expression level comparison between nonelicited and elicited cultures.**
(XLS)

Table S11 **Candidates of unknown enzymes in paclitaxel biosynthesis.**
(XLS)

Table S12 **Genes potentially involved in paclitaxel transport and expression level comparison between nonelicited and elicited cultures.**
(XLS)

Table S13 **Genes potentially involved in paclitaxel degradation and expression level comparison between nonelicited and elicited cultures.**
(XLS)

Table S14 **Comparison of genes with changes in transcript abundance after elicitation with MeJA between** ***T. × media*** **and** ***T. cuspidate*** **.**
(XLS)

Table S15 **miRNA targets and expression level comparison between nonelicited and elicited cultures.**
(XLS)

Table S16 **Primers used for qRT-PCR assays.**
(DOC)
