# Correction: Deep Sequencing Reveals Transcriptome Re-Programming of *Taxus × media* Cells to the Elicitation with Methyl Jasmonate

**DOI:** 10.1371/annotation/e57a05d7-5fd5-4ba0-a60d-64be7865fda5

**Published:** 2013-06-28

**Authors:** Guiling Sun, Yanfang Yang, Fuliang Xie, Jian-Fan Wen, Jianqiang Wu, Iain W. Wilson, Qi Tang, Hongwei Liu, Deyou Qiu

Figures 2 and 3 were incorrectly switched. 

The figure labeled as Figure 2 belongs with the title and legend for Figure 3, and the figure labeled as Figure 3 belongs with the title and legend for Figure 2. The titles and legends are in correct order. 

