# Supplementary material for: Deep Sequencing Reveals Transcriptome Re-Programming of Taxus × media Cells to the Elicitation with Methyl Jasmonate
Source: PLoS One. 2013 Apr 30;8(4):e62865. doi: 10.1371/journal.pone.0062865 (PMC3639896; doi:10.1371/journal.pone.0062865)
Supplement: Figure S1 — Taxus × media cells cultured on solid medium. (DOC) [file pone.0062865.s001.doc]

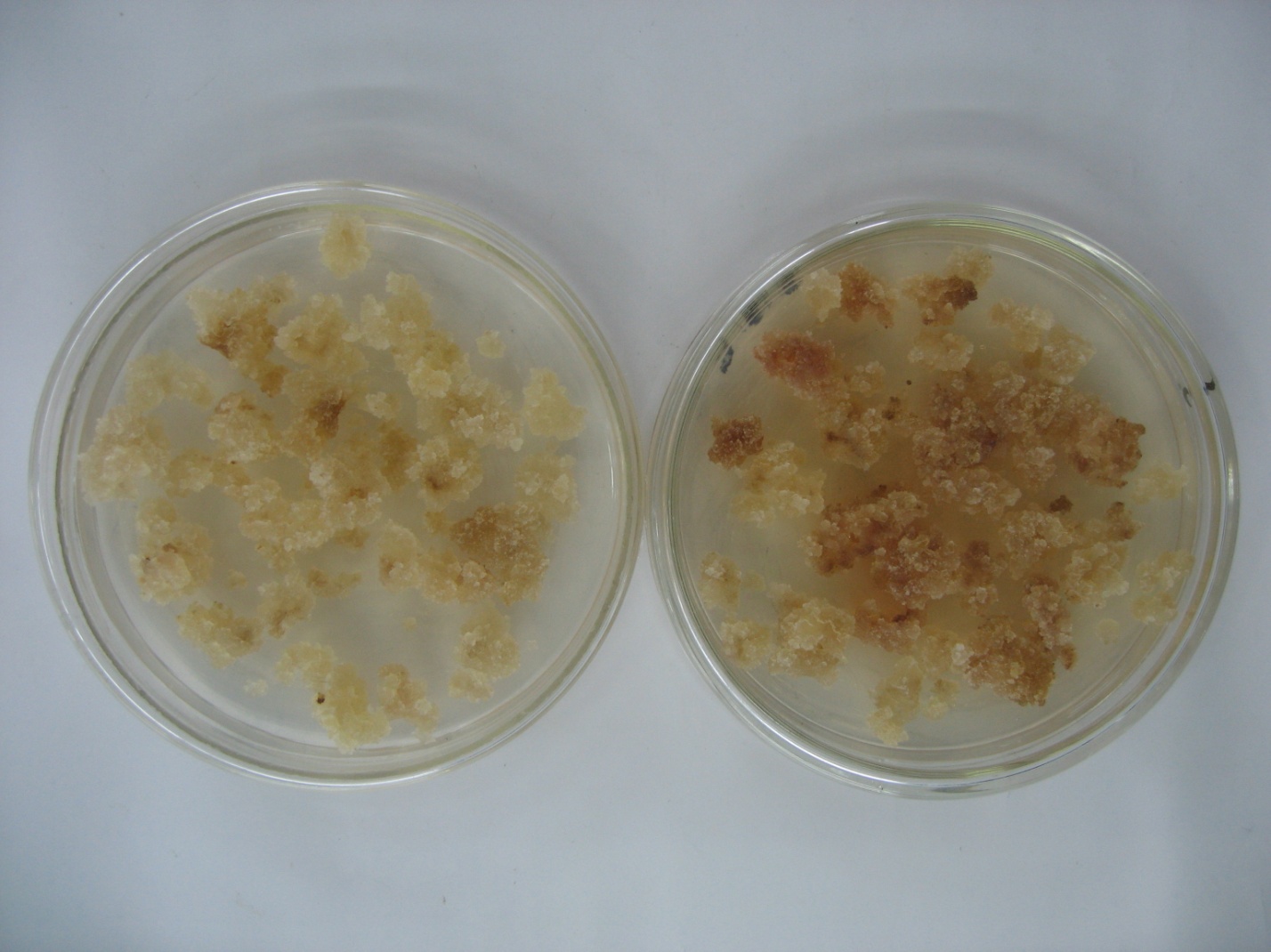


**Figure S1. *Taxus × media* cells cultured on solid medium.** Left, *T. × media* cells without elicitation of MeJA (non-elicited culture); right, *T. × media* cells after elicitation with MeJA (elicited culture).
