# Supplementary material for: Deep Sequencing Reveals Transcriptome Re-Programming of Taxus × media Cells to the Elicitation with Methyl Jasmonate
Source: PLoS One. 2013 Apr 30;8(4):e62865. doi: 10.1371/journal.pone.0062865 (PMC3639896; doi:10.1371/journal.pone.0062865)
Supplement: Figure S2 — Flow chart of paclitaxel biosynthetic pathway in T. × media . (DOC) [file pone.0062865.s002.doc]

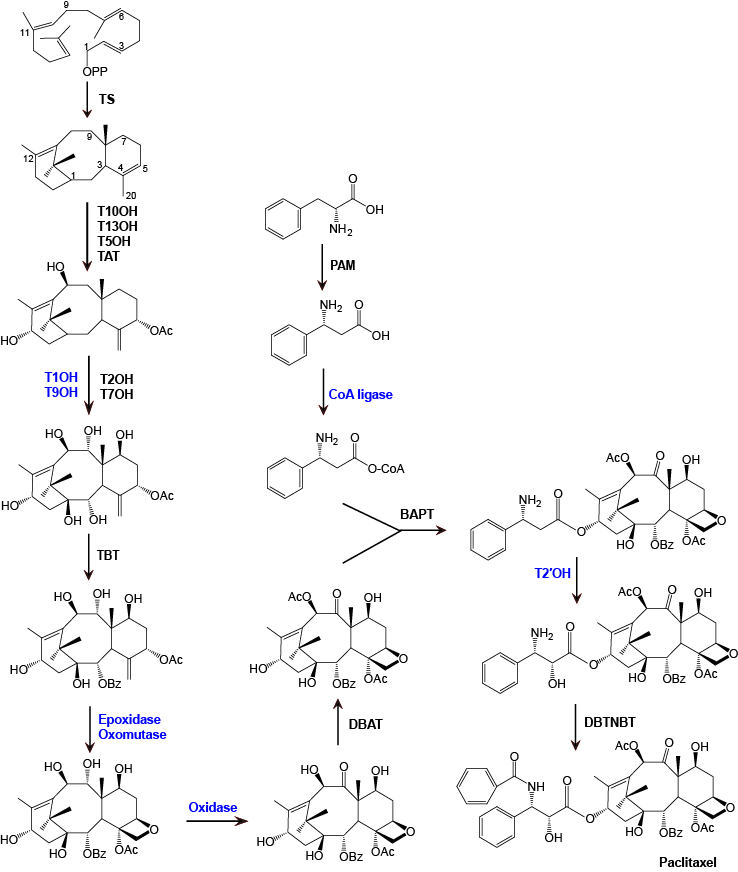


**Figure S2.** Flowchart of paclitaxel biosynthetic pathway in *T. × media*. The unknown enzymes are in blue. This flowchart is revised from Onrubia (2012).Abbreviations: BAPT: baccatin III: 3-animo-3-phenylpropanoyltransferase; DBAT: 10-deacetylbaccatin III-10-O-acetyltransferase; DBTNBT: 3′-N-debenzoyl-2′-deoxytaxol N-benzoyltransferase; PAM: phenylalanine aminomutase; TAT: taxadien-5α-ol-O-acetyl transferase; TBT: taxane 2α-O-benzoyltransferase; TS: taxa-4(5),11(12)-diene synthase; T1OH: taxane 1β-hydroxylase; T2OH: taxane 2α-hydroxylase; T2′αOH: taxane 2’α-hydroxylase; T5OH: taxadiene 5α-hydroxylase;T7OH: taxane 7β-hydroxylase; T9OH: taxane 9α-hydroxylase; T10OH: taxane 10β-hydroxylase; T13OH: taxane 13α-hydroxylase.
