# Supplementary material for: Deep Sequencing Reveals Transcriptome Re-Programming of Taxus × media Cells to the Elicitation with Methyl Jasmonate
Source: PLoS One. 2013 Apr 30;8(4):e62865. doi: 10.1371/journal.pone.0062865 (PMC3639896; doi:10.1371/journal.pone.0062865)
Supplement: Table S8 — Queries used in the BLAST search to identify genes in the main chain of terpenoid backbone biosynthesis and paclitaxel biosynthesis. (DOC) [file pone.0062865.s010.doc]

**Table S8.** Queries used in the Blast search to identify the genes involved in biosynthesis pathway of terpenoid backbone and taxol. Pathway A, 17 enzymes in the main chain of terpenoid backbone biosynthesis. Pathway B, 12 enzymes in taxol biosynthetic pathway. All the sequences are from *Taxus sp.* except those with “*”, which are from *Arabidopsis thaliana*.

| Pathway | Enzyme full name | Abbreviation | EC number | Genbank accession number |
| --- | --- | --- | --- | --- |
|  | acetyl-CoA acetyltransferase | AACT | EC 2.3.1.9 | NP_568694* |
|  | hydroxymethylglutaryl-CoA synthase | HMGS | EC 2.3.3.10 | AAT73206 |
|  | 3-hydroxy-3-methylglutaryl-coenzyme A reductase | HMGR | EC 1.1.1.34 | AAQ82685 |
|  | mevalonate kinase | MK | EC 2.7.1.36 | NP_198097* |
|  | phosphomevalonate kinase | PMK | EC 2.7.4.2 | NP_174473* |
| A | diphosphomevalonate decarboxylase | MVD | EC 4.1.1.33 | AEE79204* |
|  | 1-deoxy-D-xylulose-5-phosphate synthase | DXS | EC 2.2.1.7 | AAS89342 |
|  | 1-deoxy-D-xylulose-5-phosphate reductoisomerase | DXR | EC 1.1.1.267 | AAU87836 |
|  | 2-C-methyl-D-erythritol 4-phosphate cytidylyltransferase | MCT | EC 2.7.7.60 | ABU48538 |
|  | 4-diphosphocytidyl-2-C-methyl-D-erythritol kinase | CMK | EC 2.7.1.148 | NP_180261* |
|  | 2-C-methyl-D-erythritol 2,4-cyclodiphosphate synthase | MCS | EC 4.6.1.12 | ABU48537 |
|  | 4-hydroxy-3-methylbut-2-enyl diphosphate synthase | HDS | EC 1.17.7.1 | AED97354* |
|  | 4-hydroxy-3-methylbut-2-enyl diphosphate reductase | IDS | EC 1.17.1.2 | ABU44490 |
|  | isopentenyl-diphosphate delta-isomerase | IPI | EC 5.3.3.2 | AED92292* |
|  | geranyl diphosphate synthase | GPS | EC 2.5.1.1 | CAC16849* |
|  | farnesyl diphosphate synthase | FPS | EC 2.5.1.10 | AAS19931 |
|  | Geranylgeranyl diphosphate synthase | GGPS | EC 2.5.1.29 | AF081514 |
|  | taxa-4(5),11(12)-diene synthase | TS | EC 4.2.3.17 | AY364469 |
|  | taxadien-5α-ol-O-acetyl transferase | TAT | EC 2.3.1.162 | AF190130 |
|  | taxane 2α-O-benzoyltransferase | TBT | [EC 2.3.1.166](http://www.chem.qmul.ac.uk/iubmb/enzyme/EC2/3/1/166.html) | AF297618 |
|  | 10-deacetylbaccatin III-10-O-acetyltransferase | DBAT | EC 2.3.1.167 | AF193765 |
|  | Taxane 10-beta hydroxylase | T10OH | EC .14.13.76 | AF318211 |
| B | Taxane 13-alpha hydroxylase | T13OH | EC1.14.13.77 | AY056019 |
|  | baccatin III 3-animo-3-phenylpropanoyltransferase | BAPT |  | AY082804 |
|  | 3′-N-debenzoyl-2′-deoxytaxol N-benzoyltransferase | DBTNBT | EC [2.3.1.-](http://enzyme.expasy.org/EC/2.3.1.-) | AF466397 |
|  | Taxane 2-alpha hydroxylase | T2OH |  | AY518383 |
|  | Taxane 5-alpha hydroxylase | T5OH | EC1.14.99.37 | AY307951 |
|  | Taxane 7-beta hydroxylase | T7OH |  | AY289209 |
|  | phenylalanine aminomutase | PAM |  | AY582743 |
