# Supplementary material for: Deep Sequencing Reveals Transcriptome Re-Programming of Taxus × media Cells to the Elicitation with Methyl Jasmonate
Source: PLoS One. 2013 Apr 30;8(4):e62865. doi: 10.1371/journal.pone.0062865 (PMC3639896; doi:10.1371/journal.pone.0062865)
Supplement: Table S16 — Primers used for qRT-PCR assays. (DOC) [file pone.0062865.s018.doc]

**Table 16. Primers used for qRT-PCR assays.**

| Number | Genes | 5’-Forward Primer Sequence-3’ | 5’-Reverse Primer Sequence-3’ |
| --- | --- | --- | --- |
| 1 | TBT | AGTTTGAAGACTATACACGCCT | TACCATAGTATCCCTTTGGAAG |
| 2 | T7OH | AGATCTTCTCTCTGTTTTGCTC | CTCCAATTGCTCTTGAACTACT |
| 3 | TAT | GTTTTGTTGTGGGAGTGAGT | AAGGACGTAGCGAGTACAAG |
| 4 | DBTNBT | AGTGACTTTGCCACATAGTGTA | TTAACTGGAGATGTAGAGGGTC |
| 5 | T5OH | AGCGCATAACTATATGGTCTTC | GTGTTCAAGACCTCCTTGATT |
| 6 | DBAT | CTTGTTAGTCTACAATGCCTCA | CAAATCTCCTAAGACTGAGAGG |
| 7 | TS | ATCCTTAATCTCCTCATTGG | ATATCCCAATGGGTTGTAAGTC |
| 8 | BAPT | CTATGTGGAATTCGAAAAGC | GGTGAACCCAGAAGATTGTA |
| 9 | T10OH | CTTCTCTTGCTCTTCCGTTA | GTCCGCAATAATTGTATGGT |
| 10 | HD2 | GACAGATACACTCATCTTTCTCAG | GCAGAATATACTAGCGGTCTTT |
| 11 | ACC-1 | ATAGCAAAGGGATGTGAGACT | TCAATCTTGTCTACTGTCTTGC |
| 12 | ACC-2 | GTGTAAATATCTCCTCCTGGAA | ATGGAAACAATAGTACCGAGTG |
| 13 | CCS52a | AGAAGACCATGTTGATGAGG | GTAGGTGCATTAGCTTGGAG |
| 14 | cwf22 | CTGACAGTCTCAAATGACAGAG | CTTTTGTCATTATCTCCCTCAG |
| 15 | RAD17 | AGAGAGAAACAGATGAGATTGC | GTGACAACAGATAGACTGCAAG |
| 16 | ADH | GTACAGTCCCAAGTCTTTGTTT | ATAGTGTATCTCCTGGTGTGGT |
| 17 | 4CL | TGATGAGTTCTTTGACTCTGTC | AGGAGAAGCCCTTTTCTATAAC |
| 18 | abH | CTTGAGAAGATCACAATACTGC | TAAGAGCGCACTTTCTAGTCTC |
| 19 | CES17 | AGTCTTGTGTATTCCAGGATTC | GTCTTCACCTTTTGCCTCTAT |
| 20 | P450 | CTTTGTTGTTTGTCTGTCTCAC | TCTCTCTCCCCACATCTATTAC |
| 21 | ABC-I | ATCCTGATTATGAGGTTACTGG | ATCTCAACTGGAGACTGAAAAC |
| 22 | ABC-G | GTGCTGCTATGATACATGGTAA | TGCTATCTTAGCTATGTCGTTG |
| 23 | Actin | AAGAGAAGCTTGCTTATGTAGC | TCTGATATCCACATCACACTTC |
